# Bis(piperazine-1,4-diium) hexa­chlorido­bismuthate(III) chloride monohydrate

**DOI:** 10.1107/S1600536811045594

**Published:** 2011-11-05

**Authors:** Yu-Hua Gao, Xiao-Jia Liu, Lei Sun, Wen-Jun Le

**Affiliations:** a School of Biology and Chemical Engineering, Jiangsu University of Science and Technology, Zhenjiang 212003, People’s Republic of China

## Abstract

The crystal structure of the title compound, (C_4_H_12_N_2_)_2_[BiCl_6_]Cl·H_2_O, consists of piperazinediium cations, [BiCl_6_]^3−^ anions, Cl^−^ anions and uncoordinated water mol­ecules. The Bi^III^ cation is coordinated by six Cl^−^ anions in a slightly distorted octa­hedral geometry. The diprotonated piperazine ring adopts a chair conformation. In the crystal, extensive inter­molecular N—H⋯Cl, N—H⋯O and O—H⋯Cl hydrogen bonds occur.

## Related literature

For related structures, see: Wu *et al.* (2005 ▶); Fu *et al.* (2005 ▶)
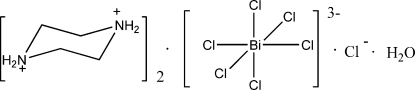


## Experimental

### 

#### Crystal data

(C_4_H_12_N_2_)_2_[BiCl_6_]Cl·H_2_O
*M* *_r_* = 651.46
Monoclinic, 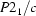

*a* = 11.085 (3) Å
*b* = 16.642 (4) Å
*c* = 11.862 (3) Å
β = 98.997 (3)°
*V* = 2161.3 (10) Å^3^
*Z* = 4
Mo *K*α radiation
μ = 9.03 mm^−1^
*T* = 296 K
0.20 × 0.20 × 0.20 mm

#### Data collection

Rigaku SCXmini diffractometer
Absorption correction: multi-scan (*CrystalClear*; Rigaku, 2005 ▶) *T*_min_ = 0.266, *T*_max_ = 0.266
12000 measured reflections
4108 independent reflections
3341 reflections with *I* > 2σ(*I*)
*R*_int_ = 0.041

#### Refinement

*R*[*F* ^2^ > 2σ(*F* ^2^)] = 0.027
*wR*(*F* ^2^) = 0.061
*S* = 1.03
4108 reflections
197 parameters
3 restraints
H atoms treated by a mixture of independent and constrained refinement
Δρ_max_ = 0.66 e Å^−3^
Δρ_min_ = −0.54 e Å^−3^

### 

Data collection: *CrystalClear* (Rigaku, 2005 ▶); cell refinement: *CrystalClear*; data reduction: *CrystalClear*; program(s) used to solve structure: *SHELXTL* (Sheldrick, 2008 ▶); program(s) used to refine structure: *SHELXTL*; molecular graphics: *SHELXTL*; software used to prepare material for publication: *SHELXTL*.

## Supplementary Material

Crystal structure: contains datablock(s) I, global. DOI: 10.1107/S1600536811045594/xu5366sup1.cif

Structure factors: contains datablock(s) I. DOI: 10.1107/S1600536811045594/xu5366Isup2.hkl

Additional supplementary materials:  crystallographic information; 3D view; checkCIF report

## Figures and Tables

**Table 1:** Hydrogen-bond geometry (Å, °)

*D*—H⋯*A*	*D*—H	H⋯*A*	*D*⋯*A*	*D*—H⋯*A*
O1—H9*A*⋯Cl2^i^	0.84 (4)	2.67 (6)	3.390 (7)	144 (6)
O1—H9*B*⋯Cl5^ii^	0.85 (6)	2.39 (6)	3.201 (6)	162 (5)
N1—H1*A*⋯Cl4^iii^	0.90	2.40	3.181 (5)	145
N1—H1*D*⋯Cl4	0.90	2.57	3.284 (5)	137
N1—H1*D*⋯Cl5	0.90	2.75	3.455 (5)	136
N2—H2*A*⋯O1	0.90	1.82	2.705 (7)	167
N2—H2*D*⋯Cl7^iv^	0.90	2.26	3.149 (5)	169
N3—H3*C*⋯Cl6	0.90	2.36	3.208 (5)	158
N3—H3*D*⋯Cl7^v^	0.90	2.21	3.069 (5)	159
N4—H4*C*⋯Cl1^vi^	0.90	2.37	3.228 (5)	158
N4—H4*D*⋯Cl4^vii^	0.90	2.43	3.155 (5)	138

Symmetry codes: (i) 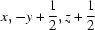; (ii) 
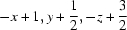
; (iii) 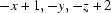; (iv) 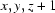; (v) 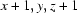; (vi) 
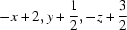
; (vii) 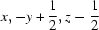.

**Fig. 1.**
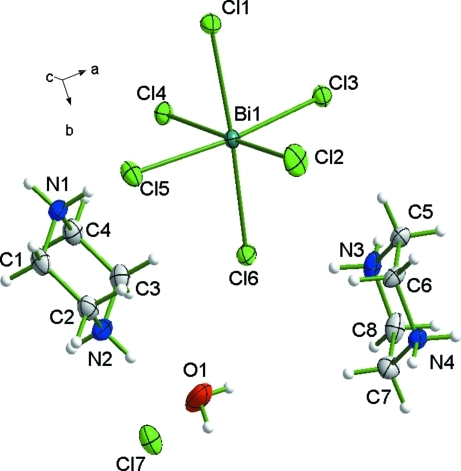
The asymmetric unit of the title compound with atom labels. Displacement ellipsoids were drawn at the 30% probability level

**Fig. 2.**
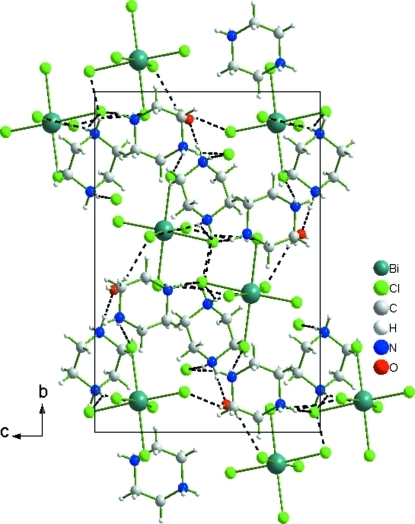
The packing viewed along the a-axis. Hydrogen bonds are drawn as dashed lines

## supplementary crystallographic information

### Comment

Recently, the crystal structure of compounds closely related to the title molecule, *e.g*., bis(piperazinium) bis(µ_2_-chloro)-octachloro-di-bismuth(iii) trihydrate (Wu *et al.*, 2005) and bis(*N*-Methylpiperazinium) bis((µ_2_-chloro)-tetrachlorobismuthate(iii))- dihydrate (Fu *et al.*, 2005) have been synthesized..We reported here thenew member of this family compounds.

The asymmetric unit of the title compound, 2C_4_H_12_N_2_^2+^.BiCl_6_^3-^.Cl^-^.H_2_O(Fig.1), consists of two piperazine cation, one [BiCl_6_]^3-^one Cl^-^anions and one water molecule. The Bi(III) ion exhibits a slightly distorted octahedral coordination environment. The diprotonated piperazine ring adopts a chair conformation. In the crystal structure, cations and anions are linked by intermolecular N—H···Cl, N—H···O and O—H···Cl hydrogen bonds into a three-dimensional network viewed along the *a*-axis (Fig.2).

### Experimental

piperazine (10 mmol, 0.86 g) BiCl_3_ (6.8 mmol, 2.15 g)and 35% aqueous HCl (3 ml) were mixed and dissolved in 30 ml water by heating to 353 K forming a clear solution. The reaction mixture was cooled slowly to room temperature, block crystals of the title compound were formed after fifteen days.

### Refinement

Water H atoms were located in a difference Fourier map and refined with O—H distance restraint of 0.85±0.01 Å, U_iso_(H) = 1.2U_eq_(O). Other H atoms were placed in calculated positions with C—H = 0.97 and N—H = 0.90 Å, and refined using a riding model with *U*_iso_(H)=1.2*U*_eq_(C,N).

### Crystal data

**Table:**

(C_4_H_12_N_2_)_2_[BiCl_6_]Cl·H_2_O	*F*(000) = 1248
*M**_r_* = 651.46	*D*_x_ = 2.002 Mg m^−^^3^
Monoclinic, *P*2_1_/*c*	Mo *K*α radiation, λ = 0.71073 Å
Hall symbol: -P 2ybc	Cell parameters from 3341 reflections
*a* = 11.085 (3) Å	θ = 1.9–26°
*b* = 16.642 (4) Å	µ = 9.03 mm^−^^1^
*c* = 11.862 (3) Å	*T* = 296 K
β = 98.997 (3)°	Block, colorless
*V* = 2161.3 (10) Å^3^	0.20 × 0.20 × 0.20 mm
*Z* = 4	

### Data collection

**Table:**

Rigaku SCXmini diffractometer	4108 independent reflections
Radiation source: fine-focus sealed tube	3341 reflections with *I* > 2σ(*I*)
graphite	*R*_int_ = 0.041
Detector resolution: 13.6612 pixels mm^-1^	θ_max_ = 26.0°, θ_min_ = 1.9°
ω scans	*h* = −13→13
Absorption correction: multi-scan (*CrystalClear*; Rigaku, 2005)	*k* = −17→20
*T*_min_ = 0.266, *T*_max_ = 0.266	*l* = −13→14
12000 measured reflections	

### Refinement

**Table:**

Refinement on *F*^2^	Secondary atom site location: difference Fourier map
Least-squares matrix: full	Hydrogen site location: inferred from neighbouring sites
*R*[*F*^2^ > 2σ(*F*^2^)] = 0.027	H atoms treated by a mixture of independent and constrained refinement
*wR*(*F*^2^) = 0.061	*w* = 1/[σ^2^(*F*_o_^2^) + (0.0226*P*)^2^] where *P* = (*F*_o_^2^ + 2*F*_c_^2^)/3
*S* = 1.03	(Δ/σ)_max_ = 0.002
4108 reflections	Δρ_max_ = 0.66 e Å^−^^3^
197 parameters	Δρ_min_ = −0.54 e Å^−^^3^
3 restraints	Extinction correction: *SHELXTL* (Sheldrick, 2008), Fc^*^=kFc[1+0.001xFc^2^λ^3^/sin(2θ)]^-1/4^
Primary atom site location: structure-invariant direct methods	Extinction coefficient: 0.00472 (14)

### Special details

**Table:**

Geometry. All e.s.d.'s (except the e.s.d. in the dihedral angle between two l.s. planes) are estimated using the full covariance matrix. The cell e.s.d.'s are taken into account individually in the estimation of e.s.d.'s in distances, angles and torsion angles; correlations between e.s.d.'s in cell parameters are only used when they are defined by crystal symmetry. An approximate (isotropic) treatment of cell e.s.d.'s is used for estimating e.s.d.'s involving l.s. planes.
Refinement. Refinement of *F*^2^ against ALL reflections. The weighted *R*-factor *wR* and goodness of fit *S* are based on *F*^2^, conventional *R*-factors *R* are based on *F*, with *F* set to zero for negative *F*^2^. The threshold expression of *F*^2^ > σ(*F*^2^) is used only for calculating *R*-factors(gt) *etc*. and is not relevant to the choice of reflections for refinement. *R*-factors based on *F*^2^ are statistically about twice as large as those based on *F*, and *R*- factors based on ALL data will be even larger.

### Fractional atomic coordinates and isotropic or equivalent isotropic displacement parameters (Å^2^)

**Table:**

	*x*	*y*	*z*	*U*_iso_*/*U*_eq_	
Bi1	0.740018 (15)	0.092026 (11)	0.807034 (15)	0.02849 (9)	
Cl2	0.79302 (15)	0.11877 (12)	0.60299 (13)	0.0670 (5)	
C6	0.9370 (5)	0.3286 (3)	0.6818 (4)	0.0390 (13)	
H6A	0.8528	0.3112	0.6758	0.047*	
H6B	0.9707	0.3059	0.6181	0.047*	
Cl3	0.97693 (11)	0.09950 (7)	0.90506 (12)	0.0394 (3)	
Cl4	0.68343 (11)	0.05928 (8)	1.02452 (11)	0.0376 (3)	
Cl6	0.70154 (12)	0.24996 (8)	0.83713 (12)	0.0442 (3)	
Cl5	0.49653 (12)	0.07440 (9)	0.74387 (13)	0.0491 (4)	
C4	0.4452 (5)	0.1743 (3)	1.0987 (5)	0.0476 (15)	
H4A	0.5070	0.1454	1.1500	0.057*	
H4B	0.3783	0.1868	1.1397	0.057*	
C3	0.4981 (5)	0.2503 (3)	1.0608 (5)	0.0459 (14)	
H3A	0.5253	0.2841	1.1265	0.055*	
H3B	0.5682	0.2380	1.0241	0.055*	
N1	0.4004 (4)	0.1236 (3)	0.9985 (4)	0.0483 (12)	
H1A	0.3673	0.0784	1.0221	0.058*	
H1D	0.4638	0.1094	0.9638	0.058*	
N2	0.4051 (4)	0.2935 (3)	0.9799 (4)	0.0485 (12)	
H2A	0.4377	0.3391	0.9570	0.058*	
H2D	0.3417	0.3069	1.0151	0.058*	
C1	0.3073 (5)	0.1661 (3)	0.9150 (5)	0.0474 (15)	
H1B	0.2357	0.1777	0.9497	0.057*	
H1C	0.2827	0.1322	0.8489	0.057*	
C2	0.3612 (5)	0.2428 (3)	0.8790 (5)	0.0474 (15)	
H2B	0.4288	0.2306	0.8388	0.057*	
H2C	0.3000	0.2719	0.8273	0.057*	
Cl1	0.76430 (12)	−0.06850 (9)	0.77947 (15)	0.0542 (4)	
N4	0.9416 (4)	0.4179 (2)	0.6760 (4)	0.0348 (10)	
H4C	1.0193	0.4338	0.6764	0.042*	
H4D	0.8970	0.4347	0.6103	0.042*	
N3	0.9625 (4)	0.3368 (3)	0.8914 (4)	0.0459 (12)	
H3C	0.8856	0.3203	0.8937	0.055*	
H3D	1.0096	0.3206	0.9561	0.055*	
C8	0.9648 (5)	0.4261 (3)	0.8847 (5)	0.0487 (15)	
H8A	1.0487	0.4443	0.8911	0.058*	
H8B	0.9301	0.4487	0.9479	0.058*	
C7	0.8937 (5)	0.4550 (3)	0.7740 (5)	0.0474 (14)	
H7A	0.8082	0.4411	0.7706	0.057*	
H7B	0.8998	0.5130	0.7695	0.057*	
C5	1.0076 (5)	0.2991 (3)	0.7909 (5)	0.0439 (14)	
H5A	1.0934	0.3118	0.7933	0.053*	
H5B	0.9998	0.2411	0.7950	0.053*	
Cl7	0.16914 (12)	0.31589 (10)	0.09392 (12)	0.0537 (4)	
O1	0.5352 (5)	0.4184 (3)	0.9159 (6)	0.099 (2)	
H9A	0.610 (3)	0.425 (4)	0.941 (6)	0.119*	
H9B	0.519 (6)	0.452 (4)	0.862 (5)	0.119*	

### Atomic displacement parameters (Å^2^)

**Table:**

	*U*^11^	*U*^22^	*U*^33^	*U*^12^	*U*^13^	*U*^23^
Bi1	0.02867 (12)	0.02880 (13)	0.02927 (13)	0.00082 (8)	0.00847 (8)	0.00086 (9)
Cl2	0.0689 (11)	0.0968 (13)	0.0406 (9)	0.0061 (9)	0.0256 (8)	0.0120 (9)
C6	0.058 (4)	0.030 (3)	0.031 (3)	−0.008 (3)	0.013 (3)	−0.001 (2)
Cl3	0.0340 (6)	0.0433 (8)	0.0412 (8)	0.0000 (6)	0.0062 (6)	0.0025 (6)
Cl4	0.0387 (7)	0.0423 (8)	0.0326 (7)	−0.0034 (6)	0.0080 (6)	0.0011 (6)
Cl6	0.0412 (7)	0.0353 (8)	0.0590 (10)	−0.0036 (6)	0.0167 (7)	−0.0065 (7)
Cl5	0.0344 (7)	0.0585 (10)	0.0538 (9)	0.0017 (6)	0.0044 (6)	−0.0146 (7)
C4	0.045 (3)	0.060 (4)	0.040 (4)	0.001 (3)	0.014 (3)	0.002 (3)
C3	0.041 (3)	0.054 (4)	0.043 (4)	−0.009 (3)	0.008 (3)	−0.011 (3)
N1	0.042 (3)	0.032 (3)	0.075 (4)	−0.007 (2)	0.021 (3)	−0.005 (3)
N2	0.057 (3)	0.035 (3)	0.059 (3)	−0.003 (2)	0.027 (3)	−0.002 (2)
C1	0.034 (3)	0.056 (4)	0.050 (4)	−0.005 (3)	0.003 (3)	−0.017 (3)
C2	0.045 (3)	0.055 (4)	0.044 (4)	0.006 (3)	0.013 (3)	0.007 (3)
Cl1	0.0409 (8)	0.0343 (8)	0.0903 (12)	0.0001 (6)	0.0192 (8)	−0.0105 (8)
N4	0.034 (2)	0.040 (3)	0.030 (3)	−0.0021 (19)	0.0023 (19)	0.008 (2)
N3	0.038 (3)	0.067 (3)	0.031 (3)	−0.014 (2)	0.000 (2)	0.019 (2)
C8	0.053 (4)	0.065 (4)	0.031 (3)	−0.018 (3)	0.015 (3)	−0.009 (3)
C7	0.053 (4)	0.044 (4)	0.047 (4)	0.001 (3)	0.016 (3)	−0.008 (3)
C5	0.044 (3)	0.040 (3)	0.049 (4)	0.000 (3)	0.012 (3)	0.012 (3)
Cl7	0.0389 (8)	0.0854 (12)	0.0366 (9)	0.0057 (7)	0.0050 (6)	−0.0004 (8)
O1	0.079 (4)	0.073 (4)	0.139 (6)	−0.016 (3)	−0.002 (4)	0.062 (3)

### Geometric parameters (Å, °)

**Table:**

Bi1—Cl2	2.6164 (16)	N2—H2D	0.9000
Bi1—Cl6	2.6954 (14)	C1—C2	1.499 (7)
Bi1—Cl5	2.7019 (14)	C1—H1B	0.9700
Bi1—Cl3	2.7036 (14)	C1—H1C	0.9700
Bi1—Cl1	2.7099 (15)	C2—H2B	0.9700
Bi1—Cl4	2.8021 (14)	C2—H2C	0.9700
C6—C5	1.488 (7)	N4—C7	1.486 (6)
C6—N4	1.488 (6)	N4—H4C	0.9000
C6—H6A	0.9700	N4—H4D	0.9000
C6—H6B	0.9700	N3—C8	1.488 (7)
C4—N1	1.479 (6)	N3—C5	1.500 (7)
C4—C3	1.493 (7)	N3—H3C	0.9000
C4—H4A	0.9700	N3—H3D	0.9000
C4—H4B	0.9700	C8—C7	1.502 (7)
C3—N2	1.480 (7)	C8—H8A	0.9700
C3—H3A	0.9700	C8—H8B	0.9700
C3—H3B	0.9700	C7—H7A	0.9700
N1—C1	1.492 (7)	C7—H7B	0.9700
N1—H1A	0.9000	C5—H5A	0.9700
N1—H1D	0.9000	C5—H5B	0.9700
N2—C2	1.483 (7)	O1—H9A	0.844 (19)
N2—H2A	0.9000	O1—H9B	0.854 (19)
			
Cl2—Bi1—Cl6	91.13 (5)	H2A—N2—H2D	108.1
Cl2—Bi1—Cl5	96.95 (5)	N1—C1—C2	109.1 (4)
Cl6—Bi1—Cl5	88.32 (4)	N1—C1—H1B	109.9
Cl2—Bi1—Cl3	92.66 (5)	C2—C1—H1B	109.9
Cl6—Bi1—Cl3	93.52 (4)	N1—C1—H1C	109.9
Cl5—Bi1—Cl3	170.17 (4)	C2—C1—H1C	109.9
Cl2—Bi1—Cl1	90.87 (6)	H1B—C1—H1C	108.3
Cl6—Bi1—Cl1	176.40 (4)	N2—C2—C1	110.5 (4)
Cl5—Bi1—Cl1	88.47 (4)	N2—C2—H2B	109.6
Cl3—Bi1—Cl1	89.38 (4)	C1—C2—H2B	109.6
Cl2—Bi1—Cl4	178.58 (5)	N2—C2—H2C	109.6
Cl6—Bi1—Cl4	90.28 (4)	C1—C2—H2C	109.6
Cl5—Bi1—Cl4	82.90 (4)	H2B—C2—H2C	108.1
Cl3—Bi1—Cl4	87.44 (4)	C7—N4—C6	111.1 (4)
Cl1—Bi1—Cl4	87.72 (5)	C7—N4—H4C	109.4
C5—C6—N4	110.7 (4)	C6—N4—H4C	109.4
C5—C6—H6A	109.5	C7—N4—H4D	109.4
N4—C6—H6A	109.5	C6—N4—H4D	109.4
C5—C6—H6B	109.5	H4C—N4—H4D	108.0
N4—C6—H6B	109.5	C8—N3—C5	111.4 (4)
H6A—C6—H6B	108.1	C8—N3—H3C	109.3
N1—C4—C3	109.9 (4)	C5—N3—H3C	109.3
N1—C4—H4A	109.7	C8—N3—H3D	109.3
C3—C4—H4A	109.7	C5—N3—H3D	109.3
N1—C4—H4B	109.7	H3C—N3—H3D	108.0
C3—C4—H4B	109.7	N3—C8—C7	110.8 (4)
H4A—C4—H4B	108.2	N3—C8—H8A	109.5
N2—C3—C4	109.9 (4)	C7—C8—H8A	109.5
N2—C3—H3A	109.7	N3—C8—H8B	109.5
C4—C3—H3A	109.7	C7—C8—H8B	109.5
N2—C3—H3B	109.7	H8A—C8—H8B	108.1
C4—C3—H3B	109.7	N4—C7—C8	110.3 (4)
H3A—C3—H3B	108.2	N4—C7—H7A	109.6
C4—N1—C1	112.0 (4)	C8—C7—H7A	109.6
C4—N1—H1A	109.2	N4—C7—H7B	109.6
C1—N1—H1A	109.2	C8—C7—H7B	109.6
C4—N1—H1D	109.2	H7A—C7—H7B	108.1
C1—N1—H1D	109.2	C6—C5—N3	110.9 (4)
H1A—N1—H1D	107.9	C6—C5—H5A	109.4
C3—N2—C2	110.7 (4)	N3—C5—H5A	109.4
C3—N2—H2A	109.5	C6—C5—H5B	109.4
C2—N2—H2A	109.5	N3—C5—H5B	109.4
C3—N2—H2D	109.5	H5A—C5—H5B	108.0
C2—N2—H2D	109.5	H9A—O1—H9B	105 (3)
			
N1—C4—C3—N2	57.7 (6)	C5—C6—N4—C7	−57.7 (5)
C3—C4—N1—C1	−57.7 (6)	C5—N3—C8—C7	55.3 (6)
C4—C3—N2—C2	−58.9 (6)	C6—N4—C7—C8	57.6 (6)
C4—N1—C1—C2	56.9 (6)	N3—C8—C7—N4	−56.3 (6)
C3—N2—C2—C1	58.8 (6)	N4—C6—C5—N3	55.9 (5)
N1—C1—C2—N2	−56.7 (6)	C8—N3—C5—C6	−55.3 (5)

### Hydrogen-bond geometry (Å, °)

**Table:**

*D*—H···*A*	*D*—H	H···*A*	*D*···*A*	*D*—H···*A*
O1—H9A···Cl2^i^	0.84 (4)	2.67 (6)	3.390 (7)	144 (6)
O1—H9B···Cl5^ii^	0.85 (6)	2.39 (6)	3.201 (6)	162 (5)
N1—H1A···Cl4^iii^	0.90	2.40	3.181 (5)	145
N1—H1D···Cl4	0.90	2.57	3.284 (5)	137
N1—H1D···Cl5	0.90	2.75	3.455 (5)	136
N2—H2A···O1	0.90	1.82	2.705 (7)	167
N2—H2D···Cl7^iv^	0.90	2.26	3.149 (5)	169
N3—H3C···Cl6	0.90	2.36	3.208 (5)	158
N3—H3D···Cl7^v^	0.90	2.21	3.069 (5)	159
N4—H4C···Cl1^vi^	0.90	2.37	3.228 (5)	158
N4—H4D···Cl4^vii^	0.90	2.43	3.155 (5)	138

Symmetry codes: (i) *x*, −*y*+1/2, *z*+1/2; (ii) −*x*+1, *y*+1/2, −*z*+3/2; (iii) −*x*+1, −*y*, −*z*+2; (iv) *x*, *y*, *z*+1; (v) *x*+1, *y*, *z*+1; (vi) −*x*+2, *y*+1/2, −*z*+3/2; (vii) *x*, −*y*+1/2, *z*−1/2.
